# Establishing bepranemab posology through exposure‐response modeling and simulation for TOGETHER, a double‐blind, placebo‐controlled Phase II study of bepranemab in prodromal–mild Alzheimer’s disease (AD)

**DOI:** 10.1002/alz70859_099277

**Published:** 2025-12-25

**Authors:** Jagdev S Sidhu, Maria Luisa Sardu, Marcus A Björnsson, Fanny Gallais, Akash Khandelwal, William Byrnes, Matthew E Barton, Irene Rebollo Mesa, Colin Ewen, Pinky Dua

**Affiliations:** ^1^ UCB, Melbourne, VIC Australia; ^2^ UCB, Milan, Milano province Italy; ^3^ Pharmetheus, Uppsala, Uppland Sweden; ^4^ Pharmetheus, Paris, Île‐de‐France France; ^5^ UCB, Monheim am Rhein, North Rhine‐Westphalia Germany; ^6^ UCB, Raleigh, NC USA; ^7^ UCB, Madrid, Madrid provincia Spain; ^8^ UCB, Slough, Berkshire United Kingdom

## Abstract

**Background:**

Bepranemab is a recombinant, humanized, full‐length immunoglobulin G4 monoclonal antibody that targets a mid‐region epitope of human tau considered essential for aggregation. TOGETHER (NCT04867616), a Phase II participant‐ and investigator‐blind, randomized, placebo‐controlled study, assessed efficacy and safety of bepranemab in people with prodromal–mild Alzheimer’s disease (AD). The relationships between clinical measures and systemic bepranemab exposure, in conjunction with placebo response, were investigated through modeling and simulation, to support bepranemab dosing for further clinical development.

**Methods:**

Pharmacokinetic, demographic, and efficacy data from a low tau burden or apolipoprotein ε4 (APOε4) genotype non‐carrier subpopulation (N=189; age range: 69.7–72 years), a “responder” population to bepranemab in the TOGETHER study (in which participants received 45 mg/kg bepranemab, 90 mg/kg bepranemab, or placebo every 4 weeks [Q4W] for <76 weeks), were modeled using non‐linear mixed‐effect models. Disease progression models, assessing Baseline scores and the influence of selected clinical covariates, were developed separately for Clinical Dementia Rating Sum of Boxes (CDR‐SB), Alzheimer's Disease Assessment Scale‐Cognitive subscale (ADAS‐Cog) 14, and tau‐positron emission tomography (PET; whole cortical gray and jack temporal meta), with each model characterizing the time‐course of placebo response and a change of response due to bepranemab exposure. Model‐based simulations were conducted to predict improvements in the 80‐week time course of each endpoint due to bepranemab dosing at 45, 60, and 90 mg/kg Q4W, versus placebo, in larger study participant cohorts with low and high tau burdens.

**Results:**

The time courses of CDR‐SB, ADAS‐Cog 14, and tau‐PET were best described by linear disease progression models. In each model, bepranemab exposure (concentrations) in all dose cohorts was found to reduce clinical markers of AD progression. For all models, simulations predicted that higher bepranemab doses (ie, >45 mg/kg) may result in improved clinical outcomes versus placebo in prodromal–mild AD.

**Conclusions:**

Exposure‐response modeling and simulation of TOGETHER study data confirmed improvement in clinical outcomes with bepranemab in a prodromal–mild AD population with low tau burden or APOε4 non‐carriers. The modeling also predicted that higher bepranemab doses are likely to be therapeutically beneficial (vs placebo) in future clinical trials.

